# One-year Safety and Effectiveness of Ustekinumab in Patients With Crohn’s Disease: The K-STAR Study

**DOI:** 10.1093/ibd/izae171

**Published:** 2024-08-03

**Authors:** Chang Kyun Lee, Won Moon, Jaeyoung Chun, Eun Soo Kim, Hyung Wook Kim, Hyuk Yoon, Hyun Soo Kim, Yoo Jin Lee, Chang Hwan Choi, Yunho Jung, Sung Chul Park, Geun Am Song, Jong Hun Lee, Eun Suk Jung, Youngdoe Kim, Su Young Jung, Jong Min Choi, Byong Duk Ye

**Affiliations:** Department of Gastroenterology, Center for Crohn’s and Colitis, Kyung Hee University Hospital, Kyung Hee University College of Medicine, Seoul, Korea; Department of Internal Medicine, Kosin University College of Medicine, Busan, Korea; Department of Internal Medicine, Gangnam Severance Hospital, Yonsei University College of Medicine, Seoul, Korea; Department of Internal Medicine, School of Medicine, Kyungpook National University, Korea; Department of Internal Medicine, Pusan National University Yangsan Hospital, Yangsan, Korea; Department of Internal Medicine, Seoul National University Bundang Hospital, Seongnam, Korea; Department of Internal Medicine, Chonnam University Medical School, Gwangju, Korea; Department of Internal Medicine, Keimyung University School of Medicine, Daegu, Korea; Department of Internal Medicine, College of Medicine, Chung-Ang University, Seoul, Korea; Department of Internal Medicine, Soonchunhyang University College of Medicine, Cheonan, Korea; Department of Internal Medicine, Kangwon National University School of Medicine, Chuncheon, Korea; Department of Internal Medicine, Pusan National University College of Medicine, Busan, Korea; Department of Internal Medicine, Dong-A University Medical Center, Busan, Korea; Medical Affairs, Janssen Korea Ltd., Seoul, Korea; Medical Affairs, Janssen Korea Ltd., Seoul, Korea; Medical Affairs, Janssen Korea Ltd., Seoul, Korea; Medical Affairs, Janssen Korea Ltd., Seoul, Korea; Department of Gastroenterology, Asan Medical Center, University of Ulsan College of Medicine, Seoul, Korea; Inflammatory Bowel Disease Center, Asan Medical Center, University of Ulsan College of Medicine, Seoul, Korea; Digestive Diseases Research Center, University of Ulsan College of Medicine, Seoul, Korea

**Keywords:** ustekinumab, Crohn’s disease, safety, effectiveness, Korea

## Abstract

**Background:**

This study investigated the safety and effectiveness of ustekinumab (UST) in Korean patients with Crohn’s disease (CD).

**Methods:**

Adult patients with CD treated with UST were prospectively enrolled in the K-STAR (Post-MarKeting Surveillance for Crohn’s Disease patients treated with STelARa) study between April 2018 and April 2022. Both the clinical effectiveness and adverse effects of UST therapy were analyzed. Missing data were handled using nonresponder imputation (ClinicalTrials.gov Identifier: NCT03942120).

**Results:**

Of the 464 patients enrolled from 44 hospitals across Korea, 457 and 428 patients (Crohn’s disease activity index ≥150) were included in the safety analysis and effectiveness analysis sets, respectively. At weeks 16 to 20 after initiating UST, clinical response, clinical remission, and corticosteroid-free remission rates were 75.0% (321 of 428), 64.0% (274 of 428), and 61.9% (265 of 428), respectively. At week 52 to 66, clinical response, clinical remission, and corticosteroid-free remission rates were 62.4% (267 of 428), 52.6% (225 of 428), and 50.0% (214 of 428), respectively. Combined effectiveness (clinical response + biochemical response) was achieved in 40.0% (171 of 428) and 41.6% (178 of 428) at week 16 to 20 and week 52 to 66, respectively. Biologic-naïve patients exhibited significantly higher rates of combined effectiveness than biologic-experienced patients (50.3% vs 30.7% at week 16-20, *P* < .001; 47.7% vs 36.0% at week 52-66, *P* = .014). No additional benefits were observed with the concomitant use of immunomodulators. Ileal location was independently associated with a higher probability of clinical remission compared with colonic or ileocolonic location at week 52 to 66. Adverse and serious adverse events were observed in 28.2% (129 of 457) and 12.7% (58 of 457), respectively, with no new safety signal associated with UST treatment.

**Conclusions:**

Ustekinumab was well-tolerated, effective, and safe as induction and maintenance therapy for CD in Korea.

**Key Messages**

**What is already known?**

Ustekinumab has been known to be effective and safe for Western patients with Crohn’s disease.

**What is new here?**

Ustekinumab induction and maintenance therapy was safe and effective for Korean patients with Crohn’s disease in the prospective, real-world, multicenter, postmarketing surveillance study.

**How can this study help patient care?**

The findings of this study add to our understanding of the role of ustekinumab in diverse patient populations with Crohn’s disease.

## Introduction

Crohn’s disease (CD) is a chronic relapsing inflammatory disorder of the gastrointestinal tract.^1^ Its onset is believed to be prompted by a combination of environmental triggers and altered gut microbiota in individuals with a particular genetic susceptibility, leading to a dysregulated host immune response.^2^ A key aspect of this immune dysregulation is the hyperactivation of the T helper type 17 (Th17) response, which is driven by interleukin (IL)-23 and plays a pivotal role in the pathogenesis of CD.^3^ Consequently, the Th17 response has emerged as a major treatment target for moderately to severely active CD, a strategy supported by several randomized controlled trials.^4–8^

Ustekinumab (UST), a human IgG1κ monoclonal antibody, binds to the p40 subunit of IL-12 and IL-23, thereby inhibiting the Th17-driven inflammatory pathway.^9^ Given its mechanism, it has been postulated that UST could effectively treat various inflammatory conditions, including CD. This hypothesis has been confirmed by the efficacy of UST against moderately to severely active psoriasis, psoriatic arthritis, ulcerative colitis, and CD, leading to the biologic’s approval for these indications.^4,10–12^ The efficacy and safety of UST in CD have been demonstrated in the long-term extension of a phase 3 clinical trial (IM-UNITI) and corroborated by several real-world studies.^13–19^ In real-world settings, the effectiveness of UST against CD at the 1-year mark has been reported as within the range of 32% to 64% for clinical remission, 32% to 51% for corticosteroid-free remission, and 16% to 39% for endoscopic remission.^14–19^

While the efficacy and safety of UST in CD have been documented, there remains a pressing need for prospective, real-world data, especially from large, diverse cohorts. Such studies are poised to answer critical clinical questions that current controlled trials have not fully addressed. For instance, since UST was introduced to the market after antitumor necrosis factor (TNF) agents and vedolizumab, an anti-integrin agent, the body of real-world evidence on UST in biologic-naïve patients is relatively limited compared with that from biologic-experienced patients. Moreover, owing to the lack of clear evidence, the combination of an immunomodulator with UST remains a common practice in real-world clinical settings. However, the effectiveness and safety of this combination therapy compared with UST monotherapy are yet to be determined. In the absence of clear clinical guidelines, the practice of combining UST with immunomodulators may persist. Therefore, we tried to provide real-life evidence on the effectiveness and safety of such combination therapy compared with UST monotherapy, contributing valuable insights for optimizing treatment strategies. In addition, the existing literature predominantly comprises retrospective studies or focuses on Western populations, leaving a knowledge gap regarding the performance UST in Asian cohorts and in real-world clinical settings beyond controlled trials.

The K-STAR (Post-MarKeting Surveillance for Crohn’s disease patients treated with STelARa) study addresses this gap by evaluating the 1-year safety and effectiveness of UST in a real-world cohort of Korean patients with CD who exhibited an inadequate response or intolerance to conventional or advanced therapies. Given that UST has been approved for both biologic-naïve and -experienced patients with CD, we aimed to offer insights into its real-world safety and effectiveness in both cohorts, particularly in Asian patients. We also explored the real-world effectiveness of combination therapy with UST and immunomodulatory therapy compared with UST monotherapy.

## Materials and Methods

### Study Population and Design

The K-STAR was a prospective, observational, multicenter, postmarketing surveillance (PMS) study. We enrolled patients with CD who were newly started on UST from 44 medical centers in Korea between April 2018 and April 2022. To clarify, patients prescribed UST for the treatment of CD, as per the product’s approval label and the physician’s clinical judgment, were eligible for our study. To be included, patients had to sign an informed consent form agreeing to the use of their personal information for PMS and be willing to participate in the PMS study.

Consequently, all patients who received at least 1 dose of UST and met the aforementioned criteria were included in our analysis. Those included in the safety analysis set had at least 1 administration record of UST. Furthermore, for the effectiveness analysis set, it consisted of participants from the safety set who also had baseline assessments including Crohn’s Disease Activity Index (CDAI) score of ≥150 and at least one follow-up effectiveness evaluation.

At the initial visit (week 0, visit 1), patients were administered an intravenous (IV) infusion of UST (260 mg for patients with a body weight ≤55 kg, 390 mg for those >55 kg to ≤85 kg, and 520 mg for those >85 kg). This initial infusion was followed by a subcutaneous (SC) injection of 90 mg of UST at the second visit (week 8), with subsequent 90 mg SC injections administered every 8 or 12 weeks for maintenance therapy. The decision-making process for determining the maintenance dose of UST in patients with CD follows the guidelines provided by the Korean approved label for UST. According to these guidelines, UST should be administered every 12 weeks (Q12W) following the induction period. However, for patients who do not demonstrate a sufficient response or who experience a loss of response to UST postinduction, an escalated dosing schedule of every 8 weeks (Q8W) is recommended. The safety and effectiveness of UST were assessed from baseline (week 0, visit 1) to any patient visit between weeks 52 and 66 (visit 5).

The study protocol was approved by the institutional review boards (IRBs) of all participating hospitals, including Asan Medical Center (IRB No. 2019-0327), and informed consent was obtained from all participating patients.

### Outcomes and Definitions

We prospectively collected the following data: patient age, sex, body mass index (BMI), smoking history, CD phenotype according to the Montreal classification,^20^ concomitant medication, history of biological exposure, prior intestinal resection, and biochemical markers such as C-reactive protein (CRP), fecal calprotectin (FC), and CDAI scores.

For the PMS study, any adverse events (AEs) and adverse drug reactions (ADRs) during UST treatment were recorded and summarized. Clinical outcomes were assessed by determining the CDAI score at visit 1 (baseline: UST IV infusion), visit 2 (first UST SC injection at week 8), visit 3 (second UST SC injection visit between weeks 16 and 20), visit 4 (third UST SC injection visit between weeks 24 and 32), and visit 5 (any visit between weeks 52 and 66). The Simple Endoscopic Score for Crohn’s Disease (SES-CD) was used to evaluate endoscopic outcomes at baseline and visit 5 (week 52-66), with biochemical markers, including serum CRP and FC levels, also analyzed. In addition, the clinical outcomes of UST monotherapy and combination therapy were compared. Ustekinumab monotherapy and combination therapy were defined as treatment with UST alone or in combination with an immunomodulator (azathioprine, methotrexate, cyclosporine, or mercaptopurine), respectively, from visits 1 to 5. Patients who either discontinued or initiated an immunomodulator during the study period were excluded from the comparative analysis of UST monotherapy and combination therapy.

An ADR was characterized as an occurrence for which the reporting physician suspected a causal relationship with UST. A serious adverse event (SAE) was defined as any untoward medical occurrence that, at any dose, results in death or persistent disability, is life-threatening, requires inpatient hospitalization, is a congenital anomaly, is a suspected transmission of any infectious agent via a medicinal product and is medically important, based on the International Council for Harmonization (ICH) and European Union (EU) Guidelines on Pharmacovigilance for Medicinal Products for Human Use.

With respect to effectiveness assessment, a clinical response was defined as a reduction of the CDAI score >70 points from baseline, and clinical remission was defined as a CDAI score <150 points.^4^ Corticosteroid-free remission was defined as clinical remission without corticosteroid use for at least 8 weeks prior to the evaluation. C-reactive protein normalization was defined as a serum level of <0.6 mg/dL, and FC normalization by a level of <250 μg/g. Combined effectiveness represented patients who achieved both a clinical and a biochemical response, as indicated by a CDAI reduction >70 points from baseline and CRP normalization (<0.6 mg/dL), respectively. Endoscopic response and remission corresponded to a >50% reduction in SES-CD score from baseline and a total SES-CD score <2, respectively. Clinically meaningful endoscopic improvement was defined as a reduction in the SES-CD score by >3 points from baseline.^21^

### Statistical Analysis

Continuous variables were presented using descriptive statistics (mean and standard deviation [SD]), whereas categorical variables were presented as frequencies with the percentage in parentheses. Subjects with 1 or more UST administration records who met the inclusion criteria were included in the safety analysis set. Safety information was reported separately as the number of subjects (with percentages), number of events, and incidence rate per 100 patient-years. Participants were included in the effectiveness assessment if they were part of the safety analysis and had undergone baseline and effectiveness evaluations during at least 1 subsequent visit.

We calculated the proportion of subjects who achieved clinical and endoscopic outcomes, including clinical response, clinical remission, corticosteroid-free remission, combined effectiveness at visits 3 (week 16-20) and 5 (week 52-66), as well as endoscopic outcomes at visit 5 (week 52-66). These outcomes were also compared with the differences between the biologic-naïve and biologic-experienced groups. The clinical outcomes and safety profiles for UST monotherapy and combination therapy were also summarized. In the evaluation of all clinical outcomes, missing data were handled using nonresponder imputation (NRI). In addition, results obtained by employing the “as-observed” or “last observation carried forward” (LOCF) imputation methodologies are included in the supplementary tables for further reference.

Where appropriate, Pearson’s χ^2^ test or Fisher’s exact test were used to compare categorical variables. Finally, univariate and multivariable logistic regression with backward selection were performed to determine the factors associated with clinical remission at visit 5 (week 52-66).

All statistical analyses were 2-sided, with *P* values < .05 indicating statistical significance. Analyses were performed using the SAS 9.4 statistical software package (Statistical Analysis System, SAS-Institute, Cary, NC, USA).

## Results

### Patient Characteristics

Of the initial 464 patients enrolled in the study (Supplementary Figure 1), 457 were eligible for inclusion in the safety analysis (median duration of follow-up 1 year [383.2 patient-years]). A total of 431 patients remained after the exclusion of individuals for whom both baseline and post-treatment effectiveness data were not available. After excluding 3 patients with a baseline CDAI <150, 428 patients were evaluated for effectiveness (Supplementary Figure 1). In the effectiveness analysis set, data were obtained from 428 patients at visit 1 (week 0), 390 patients at visit 2 (week 8), 376 patients at visit 3 (week 16-20), 292 patients at visit 4 (week 24-32), and 328 patients at visit 5 (week 52-66). The baseline characteristics of enrolled patients are summarized in Table 1.

**Table 1.** Baseline patient characteristics.

Variable	Value (N = 457)
**Sex**	
Male	305 (66.7)
Female	152 (33.3)
Age at enrollment (years)	35.0 ± 12.8
Age at the diagnosis of CD (years)	27.1 ± 12.6
Body mass index (kg/m^2^; *n* = 451)	21.4 ± 3.9
Disease duration (months; *n *= 457)	95.2 ± 77.8
**Active smoker** ^**a**^ **(*n* = 410)**	
Yes	39 (9.5)
**Disease location at baseline (*n* = 364)**	
L1, Ileum	87 (23.9)
L2, Colon	35 (9.6)
L3, Ileocolon	242 (66.5)
L4, Upper GI disease	47 (12.9)
**Disease behavior at baseline (*n* = 357)**	
B1, Nonstricturing, nonpenetrating	145 (40.6)
B2, Stricturing	141 (39.5)
B3, Penetrating	71 (19.9)
Perianal disease modifier	89 (24.9)
Crohn’s Disease Activity Index (*n *= 443)	282.1 ± 67.0
C-reactive protein at baseline (mg/dL; *n* = 374)	2.5 ± 5.2
Fecal calprotectin at baseline (μg/g; *n* = 76)	1793.1 ± 1936.4
Short Inflammatory Bowel Disease Questionnaire (SIBDQ) score (*n* = 386)	42.4 ± 12.3
**Prior exposure to biologic treatment(s) (*n* = 455)**	
Yes	243 (53.4)
Number of prior biologics (*n* = 455)	
1 biologic	146 (32.1)
2 biologics	76 (16.7)
3 biologics	21 (4.6)
**Type of biologics (*n* = 455)**	
Infliximab	179 (39.3)
Adalimumab	115 (25.3)
Vedolizumab	67 (14.7)
Infliximab + Adalimumab	61 (13.4)
Anti-TNF agent + Vedolizumab	57 (12.5)
**Prior intestinal resection(s)**	
Yes	127 (27.8)
**Concomitant medication**	
5-ASA	228 (49.9)
Systemic corticosteroids	129 (28.2)
Immunomodulators	253 (55.4)

Values are presented as Mean ± SD or number (%).

Abbreviations: 5-ASA, 5-aminosalicylic acid; anti-TNF, antitumor necrosis factor; CD, Crohn’s disease; SD, standard deviation.

^a^Patients who have ever smoked within 6 months before enrollment or who were smoking at enrollment.

Of these, 305 patients (66.7%) were male, and the average age at diagnosis was 27.1 ± 12.6 years. The mean duration from diagnosis to the initiation of UST was 95.2 ± 77.8 months. At baseline, the most common disease location was ileocolonic involvement (L3, 66.5%), and 59.4% of patients showed complicated behavior (B2, 39.5%; B3, 19.9%). The mean CDAI score stood at 282.1 ± 67.0, and the mean CRP level was 2.5 ± 5.2 mg/dL. Over half of the patients (*n* = 243, 53.4%) had been previously exposed to biologics, and 97 (21.3%) had been exposed to 2 or 3 biologics. A total of 127 (27.8%) patients had a history of intestinal resection. At the initiation of UST, 55.4% (*n* = 253) of the patients received concomitant immunomodulators. After UST induction, 45.1% of patients remained on the Q12W treatment schedule, whereas 29.4% of the patients had their dosing interval shortened to Q8W (Table S1).

### Clinical Outcomes After UST Treatment

**Figure 1.**
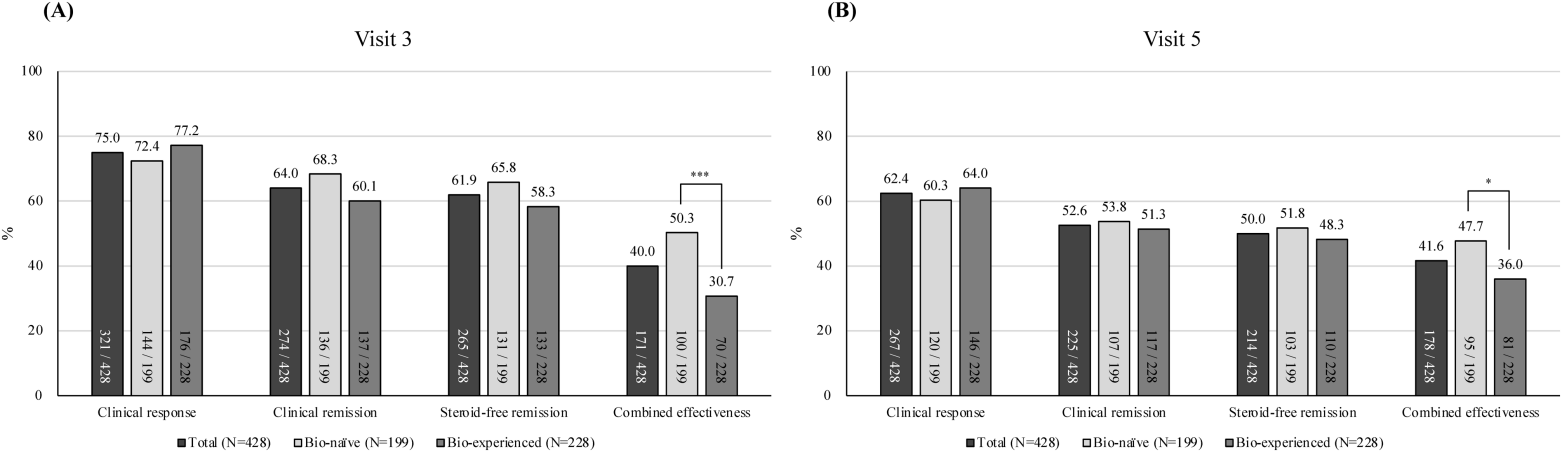
Clinical outcomes of ustekinumab treatment at (A) visit 3 (week 16-20) and (B) visit 5 (week 52-66; *P < .05, ***P < .001). The difference between the total (N = 428) and the sum of biologic-naïve and biologic-experienced (N = 427 [199 + 128]) is attributable to the absence of prior biologics use assessment for 1 patient.

The mean CDAI score at baseline was 284.7 (SD 63.1), which significantly decreased to 95.1 (SD 67.8) at visit 3 (after induction, week 16-20) and 93.3 (SD 79.4) at visit 5 (week 52-66; Table S2). At visit 3 (week 16-20), clinical response and remission were achieved in 75.0% and 64.0% of patients, respectively (Figure 1A). Similarly, at visit 5 (week 52-66), clinical response and clinical remission were achieved in 62.4% and 52.6% of patients, respectively (Figure 1B). Corticosteroid-free remission was achieved in 61.9% (95% CI, 57.3-66.5) of patients at visit 3 (week 16-20) and 50.0% (95% CI, 45.2-54.8) of patients at visit 5 (week 52-66). When combining clinical and biochemical responses (combined effectiveness), 40.0% and 41.6% of patients achieved combined effectiveness at visits 3 and 5, respectively.

As shown in Figure 1A and B, under the NRI approach, significantly higher combined effectiveness rates were observed in biologic-naïve patients than in biologic-experienced patients (50.3% vs 30.7% at visit 3, week 16-20, *P* < .001; 47.7% vs 36.0% at visit 5, week 52-66, *P* = .014). However, no significant differences were observed in clinical response, clinical remission, and corticosteroid-free remission using the NRI approach. On the other hand, rates of clinical remission, corticosteroid-free remission, and combined effectiveness at both visits 3 and 5 were significantly higher in biologic-naïve patients, according to “as observed” or LOCF imputation methodologies. Detailed information is provided in Table S3.

### Endoscopic Outcomes After UST Treatment

**Figure 2.**
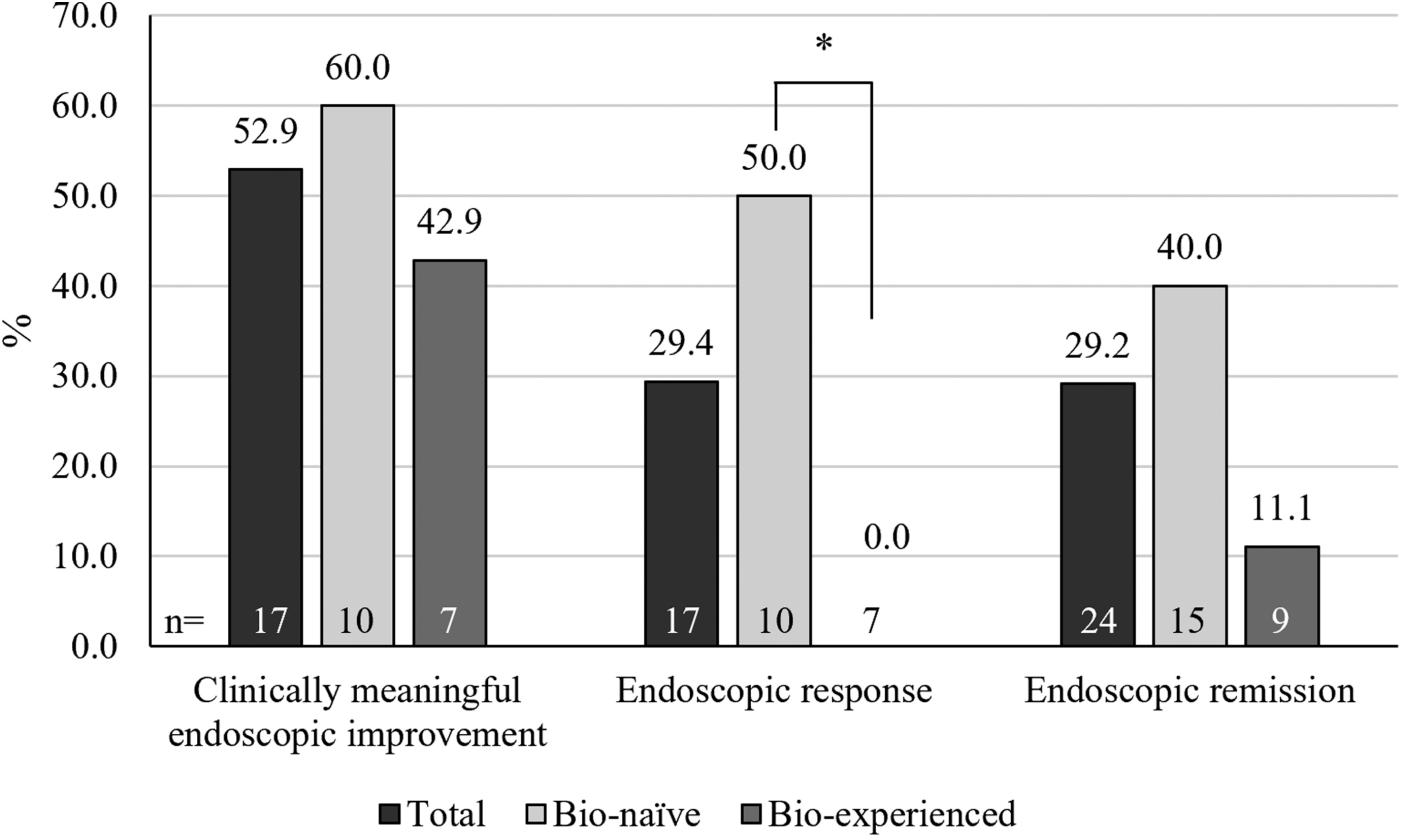
Endoscopic outcomes of ustekinumab treatment at visit 5 (week 52-66; *P < .05)

Regarding endoscopic outcomes, 42 patients underwent endoscopy at visit 1, and 24 patients underwent endoscopy at visit 5 (week 52-66). The median SES-CD score decreased from visit 1 to visit 5 (week 52-66, 13.5 [IQR 3.0-18.0] at visit 1 to 6.0 [IQR 1.0-9.0] at visit 5, week 52-66). Of the patients who underwent endoscopic evaluation at both visits (*n* = 17), 52.9% demonstrated clinically significant endoscopic improvement. Biologic-naïve patients tended to show a higher rate of endoscopic remission than their biologic-experienced counterparts, as shown in Figure 2 (40.0% vs 11.1%, *P* = .191). Furthermore, an endoscopic response was observed exclusively in biologic-naïve patients (50.0%, *P* = .044) in contrast to no response observed in any of the biologic-experienced patients.

### Comparison of UST Monotherapy vs UST in Combination With Immunomodulators

**Figure 3.**
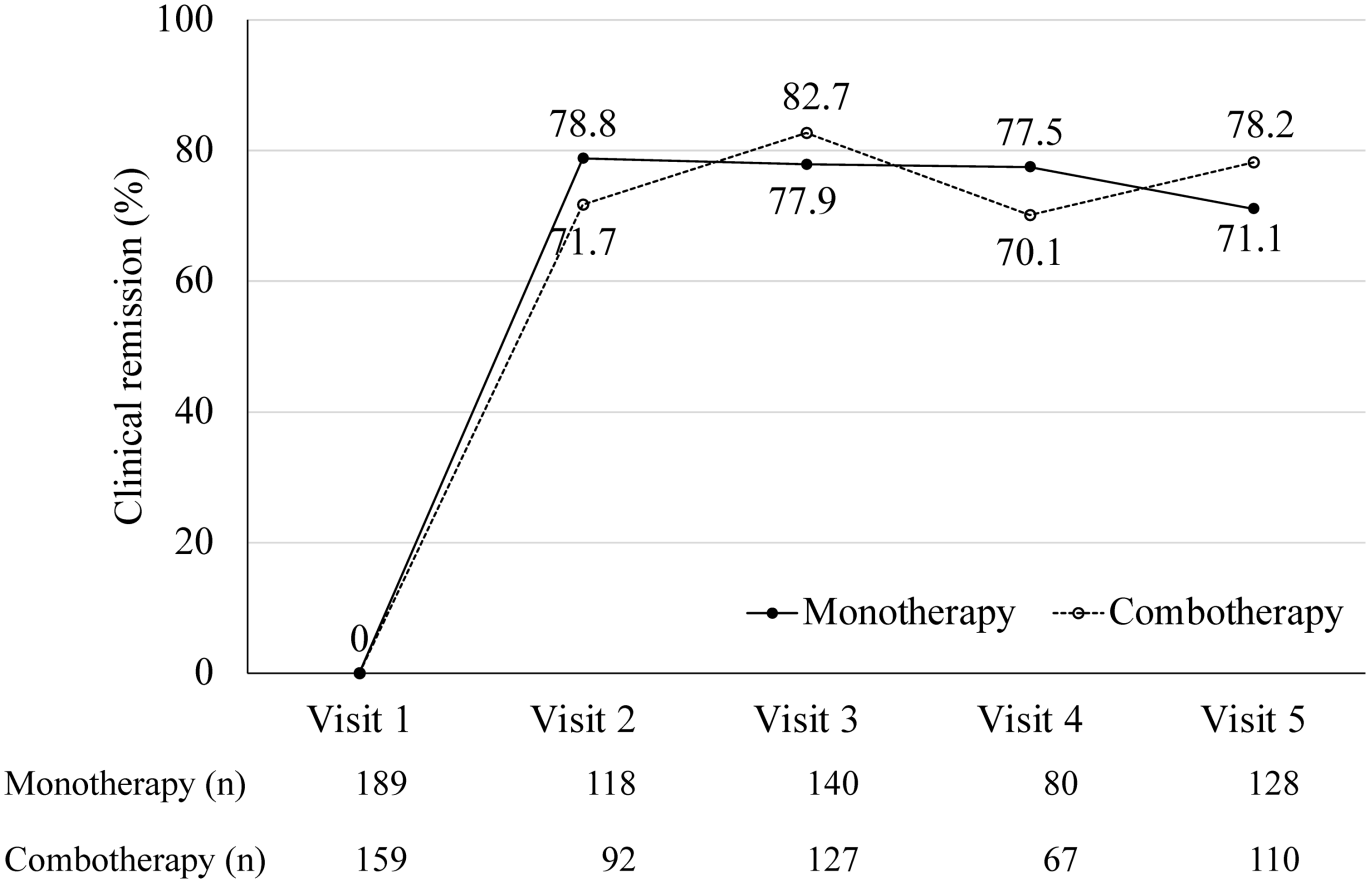
Clinical remission rates of ustekinumab monotherapy versus combination therapy with immunomodulators over the study period (as observed)

We compared patients who received UST monotherapy (*n* = 189) with those who received UST in combination with immunomodulators (combination therapy [combotherapy], *n* = 159). This analysis spanned the entire treatment period from visits 1 to 5 (see Table S4 for detailed results). As shown in Figure 3, our data suggested no discernible difference between the monotherapy and combination therapy groups, highlighting the effectiveness of UST treatment, regardless of concomitant immunomodulator use.

### Clinical Outcomes According to Baseline Disease Location and Behavior

**Figure 4.**
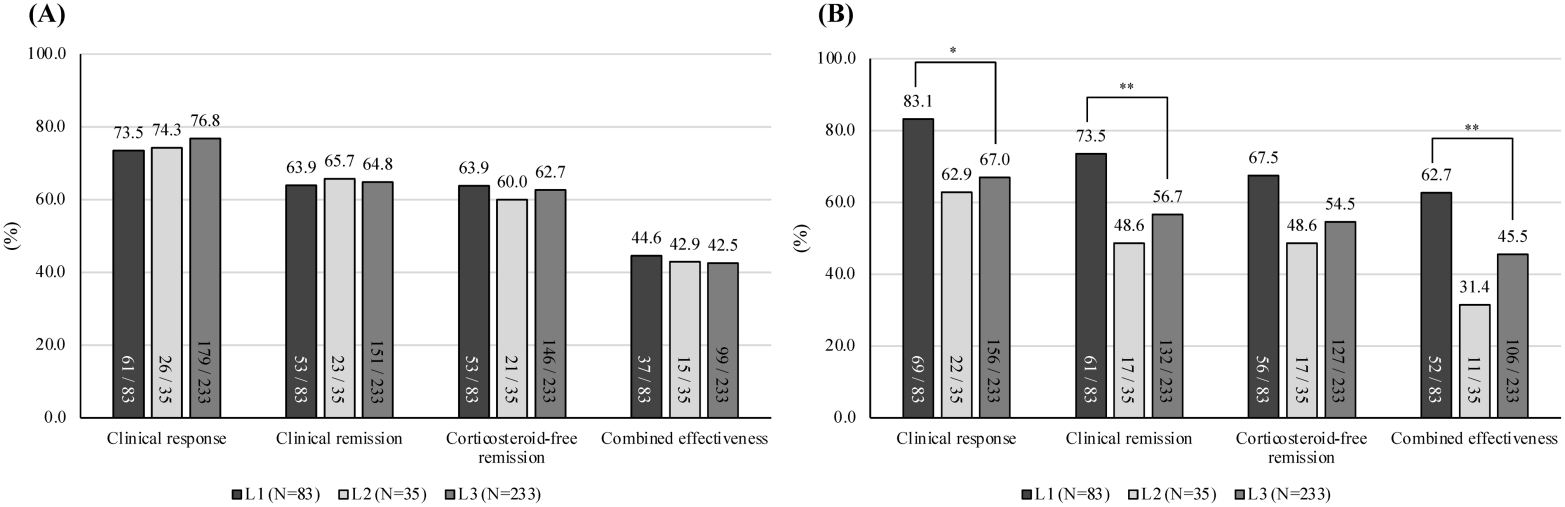
Clinical outcomes of ustekinumab treatment by disease location at (A) visit 3 (week 16-20) and (B) visit 5 (week 52-66; *P < .05, **P < .01)

Clinical outcomes including clinical response, clinical remission, and combined effectiveness at visit 3 (week 16-20) were not significantly different when categorized by disease location (L1, L2, and L3) at baseline (Figure 4A), while significant differences were observed at visit 5 (week 52-66; Figure 4B). When the outcomes of patients with L1 disease were compared with the pooled data of patients with L2 and L3 involvement at visit 5 (week 52-66), the clinical response and remission rates of patients with L1 disease were significantly higher than those of patients with L2 and L3 involvement (83.1% vs 66.4%, *P* = .004 for clinical response and 73.5% vs 55.6%, *P* = .004 for clinical remission, respectively). Similarly, the corticosteroid-free remission and combined effectiveness rates at visit 5 (week 52-66) also showed significant differences for L1 vs combined L2 and L3 (67.5% vs 53.7%, *P* = .027 for corticosteroid-free remission and 62.7% vs 43.7%, *P* = .003 for combined effectiveness, respectively; Table S5A). Furthermore, when comparing the clinical outcomes of patients according to disease behavior (B1, B2, and B3), no significant difference was observed (Table S5B).

### Changes in Biochemical Markers

There was a rapid normalization of serum CRP levels in 63.1% of patients (221 out of 350) at visit 2 (week 8) after the first IV infusion of UST (Table S6A). This normalization was maintained until visit 5. The mean CRP levels at both visits 3 and 5 were significantly lower (*P* < .001) than those at baseline (Table S6B). In the case of FC, 44.7% and 33.7% of patients showed normalized FC levels at visits 3 and 5, respectively. The mean FC levels at these visits were both significantly lower than the baseline levels (Tables S7A and S7B).

### Factors Associated With Clinical Remission at Visit 5 (week 52-66)

Based on the multivariable analysis using backward selection, we identified several factors that were inversely associated with clinical remission at visit 5 (week 52-66; Table 2). These included colonic involvement (odds ratio [OR], 0.32; 95% CI, 0.12-0.89; *P* = .028), ileocolonic involvement (OR, 0.45; 95% CI, 0.23-0.89; *P* = .021), baseline CRP levels (OR, 0.90; 95% CI, 0.84-0.97; *P* = .009), and the exposure to 2 or more biologics (OR, 0.38; 95% CI, 0.20-0.73; *P* = .003).

**Table 2.** Logistic regression analysis for predicting clinical remission at visit 5 (week 52–66).

Variable	Univariate				Multivariable		
	*n*	Odds Ratio	95% CI	*P*	Odds Ratio	95% CI	*P*
Sex = Female	428	0.87	0.59-1.30	0.509	-	-	-
Age at diagnosis							
0-16	428	Reference					
17-40		0.96	0.56-1.65	0.895	-	-	-
>40		1.66	0.80-3.42	0.174	-	-	-
Body mass index (kg/m^2^)	424	1.04	0.99-1.09	0.131	-	-	-
Disease duration (months)	428	1.00	1.00-1.00	0.241	-	-	-
Smoking status							
Active smoker = No	389	Reference					
Active smoker = Yes		1.19	0.59-2.38	0.628	-	-	-
Disease location							
L1, Ileum	351	Reference					
L2, Colon		0.34	0.15-0.78	0.010	0.32	0.12-0.89	0.028
L3, Ileocolon		0.47	0.27-0.82	0.008	0.45	0.23-0.89	0.021
L4, Upper GI disease = No	351	Reference					
L4, Upper GI disease = Yes		1.30	0.68-2.49	0.425	-	-	-
Disease behavior							
B1, Nonstricturing, nonpenetrating	345	Reference					
B2, Stricturing		1.48	0.91-2.42	0.113	-	-	-
B3, Penetrating		0.95	0.53-1.70	0.863	-	-	-
Perianal disease modifier = No	345	Reference					
Perianal disease modifier = Yes		0.63	0.39-1.03	0.066	-	-	-
C-reactive protein at baseline (mg/dL)	350	0.87	0.80-0.95	0.001	0.90	0.84-0.97	0.009
Prior biologic use							
Biologic-naïve	427	Reference					
Biologic-experienced		0.91	0.62-1.33	0.613	-	-	-
Number of prior biologics = 0	427	Reference					
Number of prior biologics = 1		1.08	0.70-1.67	0.723	0.78	0.42-1.45	0.430
Number of prior biologics ≥ 2		0.68	0.41-1.13	0.141	0.38	0.20-0.73	0.003
History of intestinal resection	428	0.99	0.65-1.51	0.977	-	-	-

Abbreviations: CI, confidence interval.

### Adverse Events

Our safety analysis included data from 457 patients exposed to UST. Of these patients, 28.2% (*n* = 129) experienced adverse events during UST treatment, corresponding to 67.9 per 100 patient-years (Table 3). Further, 7.0% of patients (*n* = 32) experienced adverse drug reactions, and 12.7% (*n* = 58) experienced serious adverse events (Table S8). Serious adverse drug reactions were observed in 10 patients (2.2%) and included abdominal pain (0.9%, 4 events), worsening of CD (0.7%, 5 events), dizziness (0.2%, 2 events), abdominal discomfort (0.2%, 1 event), anemia (0.2%, 1 event), anastomotic stenosis (0.2%, 1 event), hematochezia (0.2%, 1 event), hepatic enzyme elevation (0.2%, 1 event), pyrexia (0.2%, 1 event), and small intestinal obstruction (0.2%, 1 event). Seven events of infection in 6 patients were reported, including 2 instances of abscesses, 2 folliculitis, 2 upper respiratory tract infection, and 1 vaginal infection. However, no cases of serious infections associated with treatment were reported, and there were no cases of tuberculosis among the participants in this study. Serious adverse events and serious adverse drug reactions were observed in 14.3% and 2.1% of patients in the monotherapy group as well as in 8.2% and 1.3% of patients in the combination therapy group, respectively (Table S4B). In our study, safety concerns led to UST discontinuation in 3 patients: 1 temporarily due to concurrent sepsis and intestinal obstruction, and 2 permanently for abdominal pain and hematochezia, respectively.

**Table 3.** Summary of adverse events and adverse drug reactions during ustekinumab treatment (safety analysis set, N = 457).

Summary^a^	Incidence %	Number of Events	Incidence Rate per 100 Person-years (95% CI)
Adverse events	28.2 (129/457)	260	67.9 (63.2-72.5)
Adverse drug reactions	7.0 (32/457)	50	13.0 (9.7-16.4)
Infections			
Any	1.3 (6/457)	7^b^	1.8 (0.5-3.2)
Serious	0	0	0.0 (0.0-0.0)
Serious adverse events	12.7 (58/457)	102	26.6 (22.2-31.1)
Serious adverse drug reactions	2.2 (10/457)	18	4.7 (2.6-6.8)
Abdominal pain	0.9 (4/457)	4	1.0 (0.0-2.1)
Worsening of Crohn’s disease	0.7 (3/457)	5	1.3 (0.2-2.4)
Dizziness	0.2 (1/457)	2	0.5 (0.0-1.2)
Abdominal discomfort	0.2 (1/457)	1	0.3 (0.0-0.8)
Anemia	0.2 (1/457)	1	0.3 (0.0-0.8)
Anastomotic stenosis	0.2 (1/457)	1	0.3 (0.0-0.8)
Hematochezia	0.2 (1/457)	1	0.3 (0.0-0.8)
Hepatic enzyme increased	0.2 (1/457)	1	0.3 (0.0-0.8)
Pyrexia	0.2 (1/457)	1	0.3 (0.0-0.8)
Small intestinal obstruction	0.2 (1/457)	1	0.3 (0.0-0.8)
Death	0.0 (0/457)	0	0.0 (0.0-0.0)

^a^During 383.2 patient-years (median 1 year) follow-up.

^b^Abscess (*n* = 2), folliculitis (*n* = 2), upper respiratory tract infection (*n* = 2), and vaginal infection (*n* = 1).

Abbreviations: CI, confidence interval.

## Discussion

The K-STAR study was a real-world multicenter prospective study that explored the safety and effectiveness of UST in Korean patients with CD. Given the scarcity of real-world evidence on UST for CD in Asian populations, the current findings provide valuable insights into treatment outcomes over an approximately 1-year follow-up period. Ustekinumab was effective and well-tolerated in Korean patients, with no new or unexpected safety concerns identified. The study also confirmed the superior effectiveness of UST in biologic-naïve compared with biologic-experienced patients and found no difference in clinical remission rates between UST monotherapy and combination therapy with immunomodulators at 1 year of treatment.

The clinical outcomes observed in this study, including clinical response and remission rates, were largely consistent with the findings of the placebo-controlled UNITI trial of UST for CD.^4^ In the IM-UNITI trial, 58.1% to 59.4% of patients achieved clinical response, and 48.8% to 53.1% achieved clinical remission after 52 weeks of UST treatment, in-line with our real-world data. Although more patients achieved endoscopic remission in our study, direct comparisons are limited given the small number of patients undergoing endoscopy in the K-STAR study. Nonetheless, our effectiveness results mirror those from a recent systematic review and meta-analysis of UST observational studies by Rubín de Célix et al.^22^ This meta-analysis found clinical remission rates of 37% at 8 to 14 weeks, 42% at 16 to 24 weeks, and 45% at 48 to 52 weeks, with 33% achieving endoscopic remission in the long-term (48-52 weeks). The concordance between our real-world findings and those of previous studies underscores the effectiveness of UST in diverse patient groups, including those underrepresented in clinical trials.

Our results further confirm real-world data from East Asian countries. A recent study by Oh et al evaluated the outcomes of 65 Korean patients with CD who received UST induction therapy.^23^ They assessed clinical outcomes using CDAI scores at weeks 8 and 20, demonstrating that UST induction therapy achieved clinical response and remission in 71.4% and 55.1% of patients at week 20, respectively. Considering that the clinical response and remission rates at visit 3 (week 16-20) in our study (75% and 64%, respectively) reflect outcomes post-UST induction, our data robustly corroborate the findings of Oh et al in a considerably larger patient cohort. A separate PMS study of UST from Japan also reported its effectiveness in treating CD, without new safety concerns.^18^ In this Japanese study, the overall clinical remission rate was 49.2% at week 8 and 56.0% at week 52, further supporting the effectiveness of UST in real-world settings.

Given that UST was developed following anti-TNF agents and vedolizumab, most existing real-world studies primarily include biologic-experienced patients with CD. For instance, in the report of Oh et al, merely 10% of the patients were biologic-naïve.^23^ In our study, nearly half had not been exposed to biologics. We found significant differences in several clinical outcomes, such as combined effectiveness, via the NRI approach (50.3% for biologic-naïve vs 30.7% for biologic-experienced at visit 3, week 16-20 [*P* < .001]; and 47.7% vs 36.0% at visit 5, week 52-66 [*P* = .014]). A statistical difference was also observed in clinical remission and corticosteroid-free remission at both visits when the patient data were analyzed using the “as observed” method or when missing values were imputed using the LOCF approach. Despite the lack of significant differences in clinical response and remission between biologic-naïve and biologic-experienced patients using the NRI approach, we found that the combined effectiveness, a more stringent outcome measure, was significantly higher in biologic-naïve than in biologic-experienced patients at visit 5 (week 52-66). Thus, our data show that the former may potentially derive greater benefit from UST treatment, highlighting UST as an effective initial treatment choice for newly diagnosed or biologic-naïve patients with CD. It should, however, be noted that biologic-experienced patients with CD also benefit significantly from UST, which is recommended in the Korean clinical practice guidelines on biologics.^1^ Future studies in larger cohorts are necessary to validate these findings.

Our findings also shed light on the effectiveness of UST as a monotherapy for CD. Herein, the clinical remission rates of UST monotherapy were similar to those achieved with combination therapy, which supports the use of UST as a standalone treatment without a need for concomitant immunomodulators. An advantage of this approach is avoiding potential adverse events associated with immunomodulator use without sacrificing clinical response. This conclusion is supported by the results of previous studies. Excluding that of infliximab and thiopurines, other combinations of biological therapy and immunomodulators have yielded conflicting outcomes.^24^ A retrospective study examining CD or UC patients initiating UST therapy found that combination therapy with immunomodulators did not enhance clinical response or remission, endoscopic remission, or the persistence of UST at 1 year.^25^ Moreover, a meta-analysis found that the combination of UST with an immunomodulator did not outperform UST monotherapy in terms of inducing or maintaining remission.^26^ Therefore, our findings further support the potential use of UST as a monotherapy for the treatment of CD. However, we acknowledge that the frequent use of immunomodulators alongside UST in our cohort might have been influenced by Korean reimbursement policies requiring prior immunomodulator therapy. These policies might have resulted in a high frequency of combined UST and immunomodulator therapy. Moreover, the significant number of patients with poor prognostic indicators, including perianal disease modifier (24.9%) and prior intestinal resection (27.8%), has likely influenced physicians’ preference for ongoing immunomodulator use. Additionally, 95.9% of our bio-experienced patients (233 of 243) have previously failed anti-TNF therapy, potentially favoring combination treatment strategies. The clinical profile of CD in Korea, with more common ileal involvement, might have also guided these therapeutic choices favoring combotherapy not only for anti-TNFs but also for UST.^27,28^

It is recognized that the treatment efficacy of medications in CD may vary across different bowel segments.^29^ Consistent with this understanding, the clinical outcomes reported at week 52 to 66 in our study showed significant differences depending on disease location at baseline. That is, the clinical response, clinical remission, corticosteroid-free remission, and combined effectiveness of patients with isolated ileal disease (L1) were significantly superior to those based on the pooled data of patients with L2 and L3 (Table S5). Moreover, in the multivariable analysis (Table 2), disease location, particularly colonic and ileocolonic involvement, was found to be a significant negative predictor of clinical remission at week 52 to 66, when compared with isolated ileal involvement. This is consistent with the results from the ENEIDA registry, which also reported that ileal disease location was associated with a better response to UST treatment.^14^ Another retrospective study from Japan noted that the superiority of UST over anti-TNF biologics in managing small intestinal lesions may be due to the suppression of Th17 cells and innate lymphoid cell type 3 (ILC3) differentiation via IL-23 inhibition.^30^ Additionally, 2 previous studies on UST for the small bowel lesions also mentioned Th17 cells.^31,32^ However, given the difference in the number of patients with L1, L2, and L3 involvement in our study, these findings should be interpreted with caution and warrant further investigation in larger cohorts.

In the present study, we found that elevated baseline CRP levels and exposure to more than 2 prior biologic therapies were inversely associated with achieving clinical remission at week 52 to 66. This aligns with the findings of previous studies suggesting that a higher CRP level, which is potentially indicative of active CD, could result in a poorer response to UST.^23,33,34^ Similarly, the ENEIDA registry-based study found an association between the prior use of anti-TNF agents and the absence of clinical remission to UST therapy at both short-term (week 14) and long-term (week 52).^14,35^ Together with previous observations, our findings may help clinicians select the optimal therapy for patients with CD.

In the present study, AEs and SAEs were reported in 28.2% and 12.7% of patients, respectively. These rates are in line with previous findings, such as those from the IM-UNITI trial, where at least 1 AE was reported in 80.3 to 81.7% of the primary patient population by week 44, and SAEs occurred in 9.9 to 12.1% of patients.^4^ The IM-UNITI trial was a well-controlled study that may have reported higher rates of AEs owing to the more stringent observation compared with that in real-world studies. Notably, in our Korean patient cohort, which is considered to be at high risk for tuberculosis, we did not record any serious cases of either tuberculosis or herpes zoster infections associated with UST treatment. This finding is particularly significant and may alleviate concerns among clinicians treating patients with CD in regions with a high prevalence of these infections.

The current study had several limitations. First, due to the PMS nature of this study, which was conducted within a designated period, we encountered missing values for clinical indices and biochemical markers in some patients. This also meant that data from patients could not be followed up or collected after study termination, even if patients maintained clinical remission beyond the designated period. Second, we only included a limited number of patients who underwent endoscopy during the study period. This constrains our ability to draw broad conclusions about endoscopic outcomes, and results on endoscopic outcomes must be interpreted with caution. While colonoscopy is the gold standard for assessing endoscopic mucosal healing in Crohn’s disease, the practical constraints of noninterventional studies often limit the feasibility of such comprehensive assessments in all patients. Finally, FC was only measured in a small number of patients, meaning that FC normalization could not be included in the biochemical response of combined effectiveness. This may affect the robustness and generalizability of our conclusions regarding the effectiveness of UST in reducing intestinal inflammation. To address these limitations, future research should aim for more comprehensive data collection and larger sample sizes to validate our findings.

In conclusion, our study provides robust evidence regarding the safety and effectiveness of UST over a 1-year period in Korean patients with CD. We demonstrated its effectiveness as a monotherapy or combination therapy in both biologic-naïve and -experienced patients, suggesting favorable outcomes in the former group. The current findings add to our understanding on the potential of UST in managing patients with CD with diverse characteristics and may thus help inform clinical decisions.

## Supplementary Data

Supplementary data is available at *Inflammatory Bowel Diseases* online.

izae171_suppl_Supplementary_Material
