# Supplementary material for: One-year Safety and Effectiveness of Ustekinumab in Patients With Crohn’s Disease: The K-STAR Study
Source: Inflamm Bowel Dis. 2024 Aug 3;31(5):1306–16. doi: 10.1093/ibd/izae171 (PMC12069984; doi:10.1093/ibd/izae171)
Supplement: izae171_suppl_Supplementary_Material [file izae171_suppl_supplementary_material.docx]

**SUPPLEMENTARY FIGURE**

**Supplementary Figure 1.** Flowchart of the study population


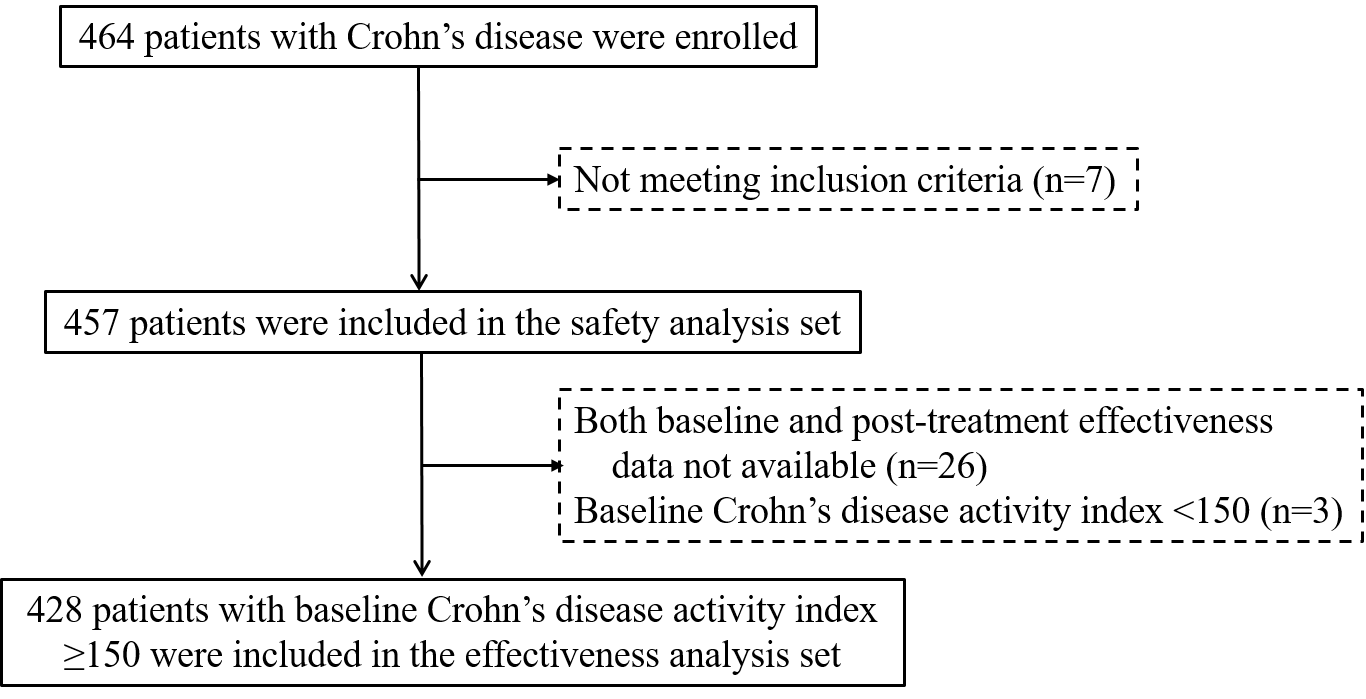


**SUPPLEMENTARY TABLES**

**Table S1.** Administration intervals of ustekinumab during the study

**Table S2.** Crohn’s disease activity index score by visit (effectiveness analysis set, N=428)

**Table S3.** Clinical outcomes of ustekinumab treatment at visit 3 (week 16–20) and visit 5 (week 52–66) (A) as observed and (B) using last observation carried forward imputation

**Table S4.** (A) Clinical outcomes of monotherapy versus combination therapy using non-responder imputation, (B) Summary of adverse events and adverse drug reactions following monotherapy versus combination therapy

**Table S5.** (A) Clinical outcomes according to disease location using non-responder imputation, (B) Clinical response/remission (effectiveness analysis set, N=428) by visit and disease behavior using non-responder imputation, (C) Clinical response/remission (effectiveness analysis set, N=428) by visit and disease behavior as observed

**Table S6.** (A) Patients (%) with C-reactive protein normalization (less than 0.6 mg/dL) by visit, (B) Serum C-reactive protein levels after ustekinumab treatment by visit

**Table S7.** (A) Patients (%) with fecal calprotectin normalization (less than 250 μg/g) by visit, (B) Fecal calprotectin levels after ustekinumab treatment by visit

**Table S8**. (A) Summary of serious adverse events (B) Summary of hospitalization events which is considered as serious adverse events

**Table S1.** Administration intervals of ustekinumab during the study

| **Dose change of administration after 1^st^ subcutaneous dose, N=428** | **n (%)** |
| --- | --- |
| Q 12 weeks | 193 (45.1) |
| Q 12 weeks–Q 8 weeks interval change | 126 (29.4) |
| Q 8 weeks | 33 (7.7) |
| Q 8 weeks–Q 12 weeks interval change | 8 (1.9) |
| More than one change of administration interval | 25 (5.8) |
| Not evaluated* | 43 (10.1) |
| *Only one subcutaneous dosing after the 1^st^ intravenous dosing | |

**Table S2.** Crohn’s disease activity index score by visit (effectiveness analysis set, N=428)

|  | | | | | | | | | | |
| --- | --- | --- | --- | --- | --- | --- | --- | --- | --- | --- |
| **Visit** | **n** | **Mean** | **SD** | **Median** | **Min** | **Max** | **95% CI** | | **P-value*** |  |
| Visit 1 (Baseline) | 415 | 284.7 | 63.1 | 268.0 | 150.0 | 766.0 | 278.7 | 290.8 |  |  |
| Visit 2 (week 8) | 251 | 106.9 | 82.5 | 92.0 | 0.0 | 433.0 | 96.6 | 117.1 |  |  |
| Visit 3 (week 16–20) | 337 | 95.3 | 67.4 | 85.0 | 0.0 | 465.0 | 88.1 | 102.5 |  |  |
| Visit 4 (week 24–32) | 185 | 108.1 | 96.3 | 87.0 | 0.0 | 517.0 | 94.2 | 122.1 |  |  |
| Visit 5 (week 52–66) | 300 | 93.6 | 79.0 | 72.5 | 0.0 | 355.0 | 84.6 | 102.6 |  |  |
| Difference (Visit 2 [week 8]-Baseline) | 242 | -171.3 | 88.3 | -180.0 | -403.0 | 116.0 | -182.5 | -160.1 | <0.0001 |  |
| Difference (Visit 3 [week 16–20]-Baseline) | 329 | -185.7 | 78.0 | -184.0 | -737.0 | 35.0 | -194.2 | -177.3 | <0.0001 |  |
| Difference (Visit 4 [week24–32]-Baseline) | 182 | -172.3 | 101.5 | -187.5 | -422.0 | 102.0 | -187.2 | -157.5 | <0.0001 |  |
| Difference (Visit 5 [week 52–66]-Baseline) | 291 | -186.6 | 83.6 | -200.0 | -488.0 | 98.0 | -196.2 | -176.9 | <0.0001 |  |
| *****Paired t-test if the variable followed a normal distribution and Wilcoxon signed-rank test if the variable did not follow a normal distribution. | | | | | | | | | | |

Abbreviations: CI, confidence interval; Max, maximum; Min, minimum; SD, standard deviation

**Table S3.** Clinical outcomes of ustekinumab treatment at visit 3 (week 16–20) and visit 5 (week 52–66) (A) as observed and (B) using last observation carried forward imputation

**(A)**

| **Effectiveness analysis set** | **Total (N=428)*** | **Biologic-naïve (n=199)** | **Biologic-experienced (n=228)** | **P-value** |
| --- | --- | --- | --- | --- |
| Clinical response, n / n' (%) | | | | |
| Visit 3 (week 16–20) | 321/329 (97.6) | 144/147 (98.0) | 176/181 (97.2) | 0.735 |
| Visit 5 (week 52–66) | 267/291 (91.8) | 120/127 (94.5) | 146/163 (89.6) | 0.132 |
| Clinical remission, n / n' (%) | | | | |
| Visit 3 (week 16–20) | 274/337 (81.3) | 136/150 (90.7) | 137/186 (73.7) | <0.001 |
| Visit 5 (week 52–66) | 225/300 (75.0) | 107/129 (83.0) | 117/170 (68.8) | 0.005 |
| Corticosteroid-free remission, n / n' (%) |  |  |  |  |
| Visit 3 (week 16–20) | 265/337 (78.6) | 131/150 (87.3) | 133/186 (71.5) | <0.001 |
| Visit 5 (week 52–66) | 214/300 (71.3) | 103/129 (79.8) | 110/170 (64.7) | 0.004 |
| Combined effectiveness, n / n’ (%) |  |  |  |  |
| Visit 3 (week 16–20) | 171/288 (59.38) | 100/128 (78.13) | 70/159 (44.03) | <0.001 |
| Visit 5 (week 52–66) | 178/282 (63.12) | 95/122 (77.87) | 82/159 (51.57) | <0.001 |

*The difference between the total (N=428) and the sum of biologic-naïve and biologic-experienced (N=427 [199+128]) is attributable to the absence of prior biologics use assessment for 1 patient.

**(B)**

| **Effectiveness analysis set** | **Total (N=428)*** | **Biologic-naïve (n=199)** | **Biologic-experienced (n=228)** | **P-value** |
| --- | --- | --- | --- | --- |
| Clinical response, n / n' (%) | | | | |
| Visit 3 (week 16–20) | 349/415 (84.1) | 164/194 (84.5) | 184/220 (83.6) | 0.803 |
| Visit 5 (week 52–66) | 360/415 (86.8) | 173/194 (89.2) | 186/220 (84.6) | 0.166 |
| Clinical remission, n / n' (%) | | | | |
| Visit 3 (week 16–20) | 299/426 (70.2) | 153/199 (76.9) | 145/226 (64.2) | 0.004 |
| Visit 5 (week 52–66) | 310/428 (72.4) | 159/199 (79.9) | 150/228 (65.8) | 0.001 |
| Corticosteroid-free remission, n / n' (%) |  |  |  |  |
| Visit 3 (week 16–20) | 268/384 (69.8) | 134/174 (77.0) | 133/209 (63.6) | 0.005 |
| Visit 5 (week 52–66) | 214/300 (71.3) | 103/129 (79.8) | 110/170 (64.7) | 0.004 |
| Combined effectiveness, n / n’ (%) |  |  |  |  |
| Visit 3 (week 16–20) | 200/397 (50.38) | 117/181 (64.64) | 82/215 (38.14) | <0.001 |
| Visit 5 (week 52–66) | 223/401 (55.61) | 126/182 (69.23) | 96/218 (44.04) | <0.001 |

*The difference between the total (N=428) and the sum of biologic-naïve and biologic-experienced (N=427 [199+128]) is attributable to the absence of prior biologics use assessment for 1 patient.

**Table S4.** (A) Clinical outcomes of monotherapy versus combination therapy using non-responder imputation, (B) Summary of adverse events and adverse drug reactions following monotherapy versus combination therapy

**(A)**

| **Visit** | **Monotherapy (N=189)** | **Combination therapy (N=159)** | **P-value** |
| --- | --- | --- | --- |
| Clinical response, n / n' (%) |  |  |  |
| Visit 3 (week 16–20) | 132/189 (69.8) | 122/159 (76.7) | 0.1494 |
| Visit 5 (week 52–66) | 115/189 (60.8) | 97/159 (61.0) | 0.9757 |
| Clinical remission, n / n' (%) |  |  |  |
| Visit 3 (week 16–20) | 109/189 (57.7) | 105/159 (66.0) | 0.1101 |
| Visit 5 (week 52–66) | 91/189 (48.2) | 86/159 (54.1) | 0.2695 |
| Corticosteroid-free remission, n / n' (%) |  |  |  |
| Visit 3 (week 16–20) | 107/189 (56.6) | 103/159 (64.8) | 0.1208 |
| Visit 5 (week 52–66) | 89/189 (47.1) | 80/159 (50.3) | 0.5488 |
| Combined effectiveness, n/n’ (%) |  |  |  |
| Visit 3 (week 16–20) | 54/189 (28.6) | 53/159 (33.3) | 0.3376 |
| Visit 5 (week 52–66) | 81/189 (42.9) | 63/159 (39.6) | 0.5417 |

**(B)**

| **Summary** | **Monotherapy (N=189)** | | | **Combination therapy (N=159)** | | |
| --- | --- | --- | --- | --- | --- | --- |
|  | **Number of events** | **Incidence %** | **Incidence rate per 100 person-years (95% CI)** | **Number of events** | **Incidence %** | **Incidence rate per 100 person-years (95% CI)** |
| Total patient-years, (median follow-up time) in years | 165.0 (1.0) | | | 135.4 (1.0) | | |
| Adverse events | 109 | 27.5 (52/189) | 66 (58.8–73.3) | 70 | 26.4 (42/159) | 51.7 (43.3–60.1) |
| Adverse drug reactions | 22 | 7.4 (14/189) | 13.3 (8.1–18.5) | 16 | 6.9 (11/159) | 11.8 (6.4–17.3) |
| Infections |  |  |  |  |  |  |
| Any | 2 | 1.1 (2/189) | 1.2 (0.0–2.9) | 5 | 2.5 (4/159) | 3.7 (0.5–6.9) |
| Serious | 0 | 0.0 (0/189) | 0.0 (0.0–0.0) | 0 | 0.0 (0/159) | 0.0 (0.0–0.0) |
| Serious adverse events | 46 | 14.3 (27/189) | 27.9 (21.0–34.7) | 14 | 8.2 (13/159) | 10.3 (5.2–15.5) |
| Serious adverse drug reactions | 7 | 2.1 (4/189) | 4.2 (1.2–7.3) | 2 | 1.3 (2/159) | 1.5 (0.0–3.5) |
| Abdominal discomfort | 0 | 0.0 (0/189) | 0.0 (0.0–0.0) | 1 | 0.6 (1/159) | 0.76 (0.0–2.2) |
| Abdominal pain | 1 | 0.5 (1/189) | 0.6 (0.0–1.8) | 1 | 0.6 (1/159) | 0.76 (0.0–2.2) |
| Anastomotic stenosis | 1 | 0.5 (1/189) | 0.6 (0.0–1.8) | 0 | 0.0 (0/159) | 0.0 (0.0–0.0) |
| Crohn's disease | 3 | 1.1 (2/189) | 1.8 (0.0–3.9) | 0 | 0.0 (0/159) | 0.0 (0.0–0.0) |
| Hematochezia | 1 | 0.5 (1/189) | 0.6 (0.0–1.8) | 0 | 0.0 (0/159) | 0.0 (0.0–0.0) |
| Small intestinal obstruction | 1 | 0.5 (1/189) | 0.6 (0.0–1.8) | 0 | 0.0 (0/159) | 0.0 (0.0–0.0) |
| Death | 0 | 0.0 (0/189) | 0.0 (0.0–0.0) | 0 | 0.0 (0/159) | 0.0 (0.0–0.0) |

Abbreviations: CI, confidence interval

**Table S5.** (A) Clinical outcomes according to disease location using non-responder imputation, (B) Clinical response/remission (effectiveness analysis set, N=428) by visit and disease behavior using non-responder imputation, (C) Clinical response/remission (effectiveness analysis set, N=428) by visit and disease behavior as observed

**(A)**

| **Visit** | **L1 (N=83)** | **Pooled data**  **L2 & L3 (N=268)** | **P-value** |
| --- | --- | --- | --- |
| Clinical response, n / n' (%) |  |  |  |
| Visit 3 (week 16–20) | 61/83 (73.5) | 205/268 (76.5) | 0.577 |
| Visit 5 (week 52–66) | 69/83 (83.1) | 178/268 (66.4) | 0.004 |
| Clinical remission, n / n' (%) |  |  |  |
| Visit 3 (week 16–20) | 53/83 (63.9) | 174/268 (64.9) | 0.859 |
| Visit 5 (week 52–66) | 61/83 (73.5) | 149/268 (55.6) | 0.004 |
| Corticosteroid-free remission, n / n' (%) | |  |  |
| Visit 3 (week 16–20) | 53/83 (63.9) | 167/268 (62.3) | 0.800 |
| Visit 5 (week 52–66) | 56/83 (67.5) | 144/268 (53.7) | 0.027 |
| Combined effectiveness, n / n' (%) | |  |  |
| Visit 3 (week 16–20) | 37/83 (44.6) | 114/268 (42.5) | 0.743 |
| Visit 5 (week 52–66) | 52/83 (62.7) | 117/268 (43.7) | 0.003 |

Abbreviations: L1, Ileum; L2, Colon, L3, Ileocolon

| **Visit** | **B1 (N=140)** | **B2 (N=137)** | **B3 (N=68)** | **P-value** |
| --- | --- | --- | --- | --- |
| Clinical response, n / n' (%) |  |  |  |  |
| Visit 3 (week 16–20) | 109/140 (77.86) | 101/137 (73.72) | 53/68 (77.94) | 0.6737 |
| Visit 5 (week 52–66) | 92/140 (65.71) | 107/137 (78.10) | 46/68 (67.65) | 0.0599 |
| Clinical remission, n / n' (%) |  |  |  |  |
| Visit 3 (week 16–20) | 95/140 (67.86) | 81/137 (59.12) | 48/68 (70.59) | 0.1728 |
| Visit 5 (week 52–66) | 80/140 (57.14) | 91/137 (66.42) | 38/68 (55.88) | 0.1940 |
| Corticosteroid-free remission, n / n' (%) |  |  |  |  |
| Visit 3 (week 16–20) | 94/140 (67.14) | 77/137 (56.20) | 46/68 (67.65) | 0.1126 |
| Visit 5 (week 52–66) | 75/140 (53.57) | 86/137 (62.77) | 38/68 (55.88) | 0.2845 |
| Corticosteroid-free: No corticosteroid use for at least 8 weeks prior to the relevant visit date | | | | |

**(B)**

Abbreviations: B1, Nonstricturing, nonpenetrating; B2, Stricturing; B3, Penetrating

**(C)**

| **Visit** | **B1 (N=140)** | **B2 (N=137)** | **B3 (N=68)** | **P-value** |
| --- | --- | --- | --- | --- |
| Clinical response, n / n' (%) |  |  |  |  |
| Visit 3 (week 16–20) | 109/111 (98.2) | 101/106 (95.28) | 53/54 (98.15) | 0.5189 |
| Visit 5 (week 52–66) | 92/99 (92.93) | 107/112 (95.54) | 46/53 (86.79) | 0.1274 |
| Clinical remission, n / n' (%) |  |  |  |  |
| Visit 3 (week 16–20) | 95/113 (84.07) | 81/108 (75.00) | 48/56 (85.71) | 0.1352 |
| Visit 5 (week 52–66) | 80/102 (78.43) | 91/115 (79.13) | 38/55 (69.09) | 0.3104 |
| Corticosteroid-free remission, n / n' (%) |  |  |  |  |
| Visit 3 (week 16–20) | 94/113 (83.19) | 77/108 (71.30) | 46/56 (82.14) | 0.0743 |
| Visit 5 (week 52–66) | 75/102 (73.53) | 86/115 (74.78) | 38/55 (69.09) | 0.7316 |

Corticosteroid-free: No corticosteroid use for at least 8 weeks prior to the relevant visit date

Abbreviations: B1, Nonstricturing, nonpenetrating; B2, Stricturing; B3, Penetrating

**Table S6.** (A) Patients (%) with C-reactive protein normalization (less than 0.6 mg/dL) by visit, (B) Serum C-reactive protein levels after ustekinumab treatment by visit

**(A)**

|  | **(%)** | **n/n'** |
| --- | --- | --- |
| Visit 1 (Baseline) | 46.3 | 162/350 |
| Visit 2 (week 8) | 63.1 | 221/350 |
| Visit 3 (week 16–20) | 61.3 | 203/331 |
| Visit 4 (week 24–32) | 56.8 | 150/264 |
| Visit 5 (week 52–66) | 66.7 | 212/318 |

**(B)**

| **Visit** | **n** | **Mean±SD** | **Median** | **Min–Max** | **Difference from baseline** |
| --- | --- | --- | --- | --- | --- |
|  |  |  |  |  | **P-value** |
| Visit 1 (Baseline) | 350 | 2.3±5.0 | 0.8 | 0.0–48.1 |  |
| Visit 2 (week 8) | 350 | 1.1±2.0 | 0.3 | 0.0–16.5 | <0.001 |
| Visit 3 (week 16–20) | 331 | 1.2±2.6 | 0.4 | 0.0–34.8 | <0.001 |
| Visit 4 (week 24–32) | 264 | 1.3±2.8 | 0.5 | 0.0–36.0 | <0.001 |
| Visit 5 (week 52–66) | 318 | 1.3±4.9 | 0.31 | 0.0–67.6 | <0.001 |

Abbreviations: Min–Max, minimum to maximum; SD, standard deviation

**Table S7.** (A) Patients (%) with fecal calprotectin normalization (less than 250 μg/g) by visit, (B) Fecal calprotectin levels after ustekinumab treatment by visit

**(A)**

|  | **(%)** | **n/n'** |
| --- | --- | --- |
| Visit 1 (Baseline) | 11.8 | 9/76 |
| Visit 3 (week 16–20) | 44.7 | 34/76 |
| Visit 5 (week 52–66) | 33.7 | 28/83 |

**(B)**

| **Visit** | **n** | **Mean±SD** | **Median** | **Min–Max** | **Difference from baseline** |
| --- | --- | --- | --- | --- | --- |
|  |  |  |  |  | **P-value** |
| Visit 1 (Baseline) | 76 | 1793.1±1936.4 | 1140 | 4.0–9390.0 |  |
| Visit 3 (week 16–20) | 76 | 1062.3±1616.1 | 384.5 | 0.0–7300.0 | <0.001 |
| Visit 5 (week 52–66) | 83 | 1108.9±1423.3 | 519 | 0.0–6000.0 | <0.001 |

Abbreviations: Min–Max, minimum to maximum; SD, standard deviation

**Table S8**. (A) Summary of serious adverse events (B) Summary of hospitalization events which is considered as serious adverse events

**(A)**

| **N=457** | **n (%)** | **Number of events** | **Incidence rate per 100 person-years (95% confidence interval)** |
| --- | --- | --- | --- |
| Total patient-years (median follow-up time) in years | 383.2 (1.0) | | |
| Serious adverse event | 58 (12.7) | 102 | 26.6 (22.2, 31.1) |
|  |  |  |  |
| Abdominal pain | 18 (3.9) | 23 | 6.0 (3.6, 8.4) |
| Crohn's disease | 8 (1.8) | 12 | 3.1 (1.4, 4.9) |
| Anal fistula | 4 (0.9) | 6 | 1.6 (0.3, 2.8) |
| Anemia | 3 (0.7) | 6 | 1.6 (0.3, 2.8) |
| Hematochezia | 5 (1.1) | 5 | 1.3 (0.2, 2.4) |
| Small intestinal obstruction | 4 (0.9) | 5 | 1.3 (0.2, 2.4) |
| Anal abscess | 2 (0.4) | 4 | 1.0 (0.0, 2.1) |
| Abdominal discomfort | 3 (0.7) | 3 | 0.8 (0.0, 1.7) |
| Intestinal obstruction | 2 (0.4) | 3 | 0.8 (0.0, 1.7) |
| Abdominal pain lower | 2 (0.4) | 2 | 0.5 (0.0, 1.2) |
| Pyrexia | 2 (0.4) | 2 | 0.5 (0.0, 1.2) |
| Dizziness | 1 (0.2) | 2 | 0.5 (0.0, 1.2) |
| Gastrointestinal hemorrhage | 1 (0.2) | 2 | 0.5 (0.0, 1.2) |
| Abdominal abscess | 1 (0.2) | 1 | 0.3 (0.0, 0.8) |
| Anastomotic stenosis | 1 (0.2) | 1 | 0.3 (0.0, 0.8) |
| Asthenia | 1 (0.2) | 1 | 0.3 (0.0, 0.8) |
| Blood pressure decreased | 1 (0.2) | 1 | 0.3 (0.0, 0.8) |
| Chills | 1 (0.2) | 1 | 0.3 (0.0, 0.8) |
| *Clostridioides difficile* infection | 1 (0.2) | 1 | 0.3 (0.0, 0.8) |
| Colonic abscess | 1 (0.2) | 1 | 0.3 (0.0, 0.8) |
| Colostomy | 1 (0.2) | 1 | 0.3 (0.0, 0.8) |
| Contusion | 1 (0.2) | 1 | 0.3 (0.0, 0.8) |
| COVID-19 pneumonia | 1 (0.2) | 1 | 0.3 (0.0, 0.8) |
| Diarrhea | 1 (0.2) | 1 | 0.3 (0.0, 0.8) |
| Enterocutaneous fistula | 1 (0.2) | 1 | 0.3 (0.0, 0.8) |
| Female genital tract fistula | 1 (0.2) | 1 | 0.3 (0.0, 0.8) |
| Gallbladder polyp | 1 (0.2) | 1 | 0.3 (0.0, 0.8) |
| Hepatic enzyme increased | 1 (0.2) | 1 | 0.3 (0.0, 0.8) |
| Ileal stenosis | 1 (0.2) | 1 | 0.3 (0.0, 0.8) |
| Intestinal hemorrhage | 1 (0.2) | 1 | 0.3 (0.0, 0.8) |
| Liver function test abnormal | 1 (0.2) | 1 | 0.3 (0.0, 0.8) |
| Mechanical ileus | 1 (0.2) | 1 | 0.3 (0.0, 0.8) |
| Melena | 1 (0.2) | 1 | 0.3 (0.0, 0.8) |
| Mesenteric abscess | 1 (0.2) | 1 | 0.3 (0.0, 0.8) |
| Pancreatitis | 1 (0.2) | 1 | 0.3 (0.0, 0.8) |
| Pelvic pain | 1 (0.2) | 1 | 0.3 (0.0, 0.8) |
| Seizure | 1 (0.2) | 1 | 0.3 (0.0, 0.8) |
| Sepsis | 1 (0.2) | 1 | 0.3 (0.0, 0.8) |
| Small intestinal perforation | 1 (0.2) | 1 | 0.3 (0.0, 0.8) |
| Vulvovaginal injury | 1 (0.2) | 1 | 0.3 (0.0, 0.8) |

**(B)**

| **N=457** | **n (%)** | **Number of events** | **Incidence rate per 100 person-years (95% confidence interval)** |
| --- | --- | --- | --- |
| Total patient-years (median follow-up time) in years | 383.2 (1.0) | | |
| Hospitalization | 56 (12.3) | 96 | 25.1 (20.7, 29.4) |
|  |  |  |  |
| Abdominal pain | 18 (3.9) | 23 | 6.0 (3.6, 8.4) |
| Crohn's disease | 8 (1.8) | 12 | 3.1 (1.4, 4.9) |
| Anal fistula | 4 (0.9) | 6 | 1.6 (0.3, 2.8) |
| Anemia | 3 (0.7) | 6 | 1.6 (0.3, 2.8) |
| Hematochezia | 5 (1.1) | 5 | 1.3 (0.2, 2.4) |
| Small intestinal obstruction | 4 (0.9) | 5 | 1.3 (0.2, 2.4) |
| Anal abscess | 2 (0.4) | 4 | 1.0 (0.0, 2.1) |
| Abdominal discomfort | 3 (0.7) | 3 | 0.8 (0.0, 1.7) |
| Intestinal obstruction | 2 (0.4) | 3 | 0.8 (0.0, 1.7) |
| Abdominal pain lower | 2 (0.4) | 2 | 0.5 (0.0, 1.2) |
| Pyrexia | 2 (0.4) | 2 | 0.5 (0.0, 1.2) |
| Dizziness | 1 (0.2) | 2 | 0.5 (0.0, 1.2) |
| Gastrointestinal hemorrhage | 1 (0.2) | 2 | 0.5 (0.0, 1.2) |
| Abdominal abscess | 1 (0.2) | 1 | 0.3 (0.0, 0.8) |
| Anastomotic stenosis | 1 (0.2) | 1 | 0.3 (0.0, 0.8) |
| Asthenia | 1 (0.2) | 1 | 0.3 (0.0, 0.8) |
| Chills | 1 (0.2) | 1 | 0.3 (0.0, 0.8) |
| *Clostridioides difficile* infection | 1 (0.2) | 1 | 0.3 (0.0, 0.8) |
| Colonic abscess | 1 (0.2) | 1 | 0.3 (0.0, 0.8) |
| Colostomy | 1 (0.2) | 1 | 0.3 (0.0, 0.8) |
| Contusion | 1 (0.2) | 1 | 0.3 (0.0, 0.8) |
| COVID-19 pneumonia | 1 (0.2) | 1 | 0.3 (0.0, 0.8) |
| Diarrhea | 1 (0.2) | 1 | 0.3 (0.0, 0.8) |
| Enterocutaneous fistula | 1 (0.2) | 1 | 0.3 (0.0, 0.8) |
| Female genital tract fistula | 1 (0.2) | 1 | 0.3 (0.0, 0.8) |
| Gallbladder polyp | 1 (0.2) | 1 | 0.3 (0.0, 0.8) |
| Ileal stenosis | 1 (0.2) | 1 | 0.3 (0.0, 0.8) |
| Intestinal hemorrhage | 1 (0.2) | 1 | 0.3 (0.0, 0.8) |
| Mechanical ileus | 1 (0.2) | 1 | 0.3 (0.0, 0.8) |
| Melaena | 1 (0.2) | 1 | 0.3 (0.0, 0.8) |
| Pelvic pain | 1 (0.2) | 1 | 0.3 (0.0, 0.8) |
| Seizure | 1 (0.2) | 1 | 0.3 (0.0, 0.8) |
| Small intestinal perforation | 1 (0.2) | 1 | 0.3 (0.0, 0.8) |
| Vulvovaginal injury | 1 (0.2) | 1 | 0.3 (0.0, 0.8) |
